# Bioinformatics analysis of long non-coding RNA-associated competing endogenous RNA network in schizophrenia

**DOI:** 10.1038/s41598-021-03993-3

**Published:** 2021-12-24

**Authors:** Hani Sabaie, Madiheh Mazaheri Moghaddam, Marziyeh Mazaheri Moghaddam, Noora Karim Ahangar, Mohammad Reza Asadi, Bashdar Mahmud Hussen, Mohammad Taheri, Maryam Rezazadeh

**Affiliations:** 1 Molecular Medicine Research Center, Tabriz University of Medical Sciences, Tabriz, Iran; 2 Department of Medical Genetics, Faculty of Medicine, Tabriz University of Medical Sciences, Tabriz, Iran; 3 Department of Genetics and Molecular Medicine, School of Medicine, Zanjan University of Medical Sciences (ZUMS), Zanjan, Iran; 4 Immunology Research Center, Tabriz University of Medical Sciences, Tabriz, Iran; 5 Student Research Committee, Tabriz University of Medical Sciences, Tabriz, Iran; 6 Department of Pharmacognosy, College of Pharmacy, Hawler Medical University, Kurdistan Region, Erbil, Iraq; 7 Men’s Health and Reproductive Health Research Center, Shahid Beheshti University of Medical Sciences, Tehran, Iran

**Keywords:** Computational biology and bioinformatics, Genetics, Psychology

## Abstract

Schizophrenia (SCZ) is a serious psychiatric condition with a 1% lifetime risk. SCZ is one of the top ten global causes of disabilities. Despite numerous attempts to understand the function of genetic factors in SCZ development, genetic components in SCZ pathophysiology remain unknown. The competing endogenous RNA (ceRNA) network has been demonstrated to be involved in the development of many kinds of diseases. The ceRNA hypothesis states that cross-talks between coding and non-coding RNAs, including long non-coding RNAs (lncRNAs), via miRNA complementary sequences known as miRNA response elements, creates a large regulatory network across the transcriptome. In the present study, we developed a lncRNA-related ceRNA network to elucidate molecular regulatory mechanisms involved in SCZ. Microarray datasets associated with brain regions (GSE53987) and lymphoblasts (LBs) derived from peripheral blood (sample set B from GSE73129) of SCZ patients and control subjects containing information about both mRNAs and lncRNAs were downloaded from the Gene Expression Omnibus database. The GSE53987 comprised 48 brain samples taken from SCZ patients (15 HPC: hippocampus, 15 BA46: Brodmann area 46, 18 STR: striatum) and 55 brain samples taken from control subjects (18 HPC, 19 BA46, 18 STR). The sample set B of GSE73129 comprised 30 LB samples (15 patients with SCZ and 15 controls). Differentially expressed mRNAs (DEmRNAs) and lncRNAs (DElncRNAs) were identified using the limma package of the R software. Using DIANA-LncBase, Human MicroRNA Disease Database (HMDD), and miRTarBase, the lncRNA- associated ceRNA network was generated. Pathway enrichment of DEmRNAs was performed using the Enrichr tool. We developed a protein–protein interaction network of DEmRNAs and identified the top five hub genes by the use of STRING and Cytoscape, respectively. Eventually, the hub genes, DElncRNAs, and predictive miRNAs were chosen to reconstruct the subceRNA networks. Our bioinformatics analysis showed that twelve key DEmRNAs, including *BDNF*, *VEGFA*, *FGF2*, *FOS*, *CD44*, *SOX2*, *NRAS*, *SPARC*, *ZFP36*, *FGG*, *ELAVL1*, and *STARD13*, participate in the ceRNA network in SCZ. We also identified *DLX6-AS1*, *NEAT1*, *MINCR*, *LINC01094*, *DLGAP1-AS1*, *BABAM2-AS1*, *PAX8-AS1*, *ZFHX4-AS1*, *XIST*, and *MALAT1* as key DElncRNAs regulating the genes mentioned above. Furthermore, expression of 15 DEmRNAs (e.g., *ADM* and *HLA-DRB1*) and one DElncRNA (*XIST*) were changed in both the brain and LB, suggesting that they could be regarded as candidates for future biomarker studies. The study indicated that ceRNAs could be research candidates for investigating SCZ molecular pathways.

## Introduction

Schizophrenia (SCZ) is a severe psychotic disorder with a lifetime risk of almost 1%. SCZ is considered one of the ten most common causes of disability throughout the world^1^. The symptoms of SCZ are usually classified into three categories: positive symptoms such as hallucinations, delusions, and thinking problems; negative symptoms such as diminished emotions, reduced motivation, decreased speaking, and social deterioration; cognitive symptoms such as attention, emphasis, processing speed, and memory dysfunctions^1,2^. The etiology of SCZ is demonstrated to be multifactorial, in which both environmental and genetic factors have been identified to participate in the manifestation of symptoms^3^. Dysregulated gene expression and protein production are considered to be associated with the pathophysiology of SCZ and have spatial variation across brain regions and temporal variation during disease progression^4–6^. A recent postmortem transcriptional profiling study shows a considerable burden of differentially expressed genes (DEGs) across hippocampus (HPC), Brodmann area 46 (BA46), and striatum (STR) regions and reveals that multiple signaling and inflammatory pathways are dysregulated in all three regions in SCZ^7^. On the other hand, a growing body of evidence has indicated that the brain is linked to the periphery via the circulatory system containing hormones and other secreted regulatory molecules produced in the diffuse neuroendocrine system which impact the gene expression pattern of the peripheral markers^8,9^. These findings are confirmatory evidence that SCZ is a systemic disorder and support the notion that investigation of the SCZ can be through studying the biomarkers in the peripheral samples such as whole blood, peripheral blood mononuclear cells (PBMCs), lymphoblasts (LBs), and olfactory epithelium^10–13^. Numerous studies demonstrated alterations in non-coding RNA (ncRNA) in people with SCZ^14^. These findings provided information concerning the underlying mechanisms of dysregulation in gene expression and protein production. The ncRNA entails several RNA molecule families with a genetic distinction based on mature sequence size^14^. A growing body of evidence suggests that microRNAs (miRNAs) (20–22 nucleotides) are aberrantly expressed in the brains of SCZ patients^15,16^. Furthermore, new research indicates that the aberrant expression of other ncRNAs, including long non-coding RNAs (lncRNAs) with more than 200 nucleotides, could be implicated in SCZ^17,18^.

Remarkable data have been provided about the molecular mechanisms and biological procedures of SCZ through advanced genetic, genomic, developmental neurobiology, and systems biology investigations. High-throughput discovery of genes that contribute to the molecular pathology of SCZ is now possible using microarray technologies and next-generation sequencing^7,13,19,20^. The role of genetic in SCZ pathophysiology is still vague, and newly discovered mechanisms responsible for regulating gene transcription could be beneficial in resolving how these alterations cause the development and progression of SCZ. As a result, potencies for more efficient treatment and diagnosis are found. Pier Paolo Pandolfi and colleagues recommended a novel regulatory mechanism, known as competing endogenous RNA (ceRNA), in 2011^21^. It was suggested that cross-talk between both coding and non-coding RNAs such as lncRNAs, circular RNAs (circRNAs), and pseudogenes via miRNA response elements (MREs), as miRNA complementary sequences, leads to the formation of a large-scale regulatory network in diverse parts of the transcriptome^22^. According to the ceRNA hypothesis, RNAs transcripts can regulate each other by competing for shared miRNAs. In addition, the expression levels of these two RNA transcripts will have a positive relationship with each other^21^. Over the last few years, numerous studies have validated the ceRNA theory. It is well known that disruption of the balance of ceRNA cross-talk is associated with various developmental processes and pathological conditions, including tumorigenesis, neurodegenerative diseases (e.g., Alzheimer’s disease (AD))^23^, and mental disorders (e.g., SCZ and depression)^24,25^.

Given that SCZ is one of the ten most prevalent causes of disability globally and since the nature of the genetic component in SCZ pathophysiology is not fully understood, we performed a bioinformatics analysis to elucidate the lncRNA-associated ceRNA network to clarify molecular regulatory mechanisms involved in SCZ.

## Methods

In the present study, we employed a system biology approach to mine data of the two microarray datasets of multiple regions of the brain (GSE53987) and the LBs derived from peripheral blood (sample set B from GSE73129) from subjects with SCZ and matched controls. We intended to recognize differentially expressed mRNAs (DEmRNAs) and lncRNAs (DElncRNAs) and make a lncRNA-associated ceRNA network. All methods were carried out in accordance with relevant guidelines and regulations.

### Gene expression profile data collection

The gene expression profiles mentioned above were obtained from the NCBI Gene Expression Omnibus database (GEO, https://www.ncbi.nlm.nih.gov/geo/). A chip-based platform GPL570 (HG-U133_Plus_2) Affymetrix Human Genome U133 Plus 2.0 Array was applied for both datasets. The GSE53987 included 48 brain samples from SCZ patients (15 HPC, 15 BA46, 18 STR) and 55 brain samples from control subjects (18 HPC, 19 BA46, 18 STR)^7^. The sample set B from GSE73129 contained 30 LB samples, of which 15 were SCZ patients, and 15 were control^13^.

### Data preprocessing and DEmRNAs and DElncRNAs identification

We used Robust Multichip Average (RMA) for background correction, and quantile normalization of all the raw data files^26^. Brain regions and LB samples were normalized separately. An interquartile range (IQR) filter (IQR across the samples on the log base two scale greater than median IQR) was used to reduce the number of evaluated genes followed by an intensity filter (a minimum of > 100 expression signals in a minimum of 25% of the arrays) aiming to remove the non-significant probe sets which are not expressed or not changing^27^. AgiMicroRna Bioconductor package was used for assessing the quality control^28^. We employed principal component analysis (PCA) to conduct a dimensional reduction analysis^29^, aiming to find similarities between each sample group using R software’s ggplot2 package^30^. Besides, a heatmap was drawn to show the correlation between samples using the Pheatmap package of R^31^. Differential expression gene analysis (DEGA) was done between SCZ and normal samples using the linear models for microarray data (limma) R package^32^ in Bioconductor (https://www.bioconductor.org/)^33^. We used the removeBatchEffect() function from the limma package to remove batch effects. Age, gender, and race were used as covariates in GSE73129 study and race and post-mortem interval in GSE53987 study to modify the data for confounding variables. We did a linear model on the main factor “group” (disease vs. control) using limma’s lmFit() function and included covariates in the linear model as well. The limma’s eBayes () function was then used to calculate differentially expressed data from the linear fit model. We utilized the previously used approach to identify lncRNA probes^34^. We downloaded the complete list of lncRNA genes with approved HUGO Gene Nomenclature Committee (HGNC) symbols from (https://www.genenames.org/)^35^. Then, we compared the lncRNA gene list with our dataset gene symbols and chose the overlapped genes. Student t-test was used and we set the aberrantly expressed RNAs cut-off as: (1) a false discovery rate (adjusted *P*-value) < 0.001, and (2) |log2 fold change (log2FC)|> 0.69. Note that we discarded probes with no gene symbols.

### lncRNA-associated ceRNA network construction

DIANA-LncBase v3^36^ was used to identify the experimentally validated interactions between the miRNAs and lncRNAs. *Homo Sapiens* “Species”, Brain or Peripheral Blood “Tissue”, and high “miRNA Confidence Levels” were selected as criteria for the DIANA-LncBase query. SCZ-related miRNAs were collected from the Human microRNA Disease Database (HMDD) v3.2 database^37^. Besides, we obtained the interactions between miRNAs that were collected using the HMDD database and target mRNAs supported by strong experimental evidence from miRTarBase^38^. These mRNAs were then compared with the previously obtained mRNAs. Then, we used the duplicated mRNAs to construct the lncRNA-miRNA-mRNA network. LncRNAs, targeted mRNAs, and the interacted miRNAs were removed from the ceRNA network in the opposite expression pattern between the targeted mRNAs and lncRNAs. Cytoscape software (version 3.8.0)^39^ was used to generate ceRNA regulatory network.

### Pathway enrichment analysis of DEmRNAs

The Kyoto Encyclopedia of Genes and Genomes (KEGG) enrichment analysis was performed using the Enrichr tool^40,41^ for pathway enrichment to analyze the DEmRNAs that were in the ceRNA network.

### Protein–protein interaction (PPI) network construction and hub genes identification

A PPI network was constructed using online STRING database (https://string-db.org/)^42^ for predicting the interactive relationships of common DEmRNAs encoding proteins. A minimum interaction score of above 0.7 (high confidence) was needed for PPI network construction. Cytoscape software was employed for PPI network visualization and hub genes analysis. For PPI network simplification, we removed the non-interacting genes. Then, we considered the top five genes with the highest degree of connection to the others as the hub genes according to CytoHubba (version 0.1) analysis^43^ from Cytoscape.

### Reconstruction of the subceRNA network

We retrieved the corresponding lncRNA and miRNA from the original network of ceRNA. We used them in the reconstruction of the subceRNA network to confirm the ceRNA network of hub genes.

## Results

### DEmRNAs and DElncRNAs identification

**Figure 1:**
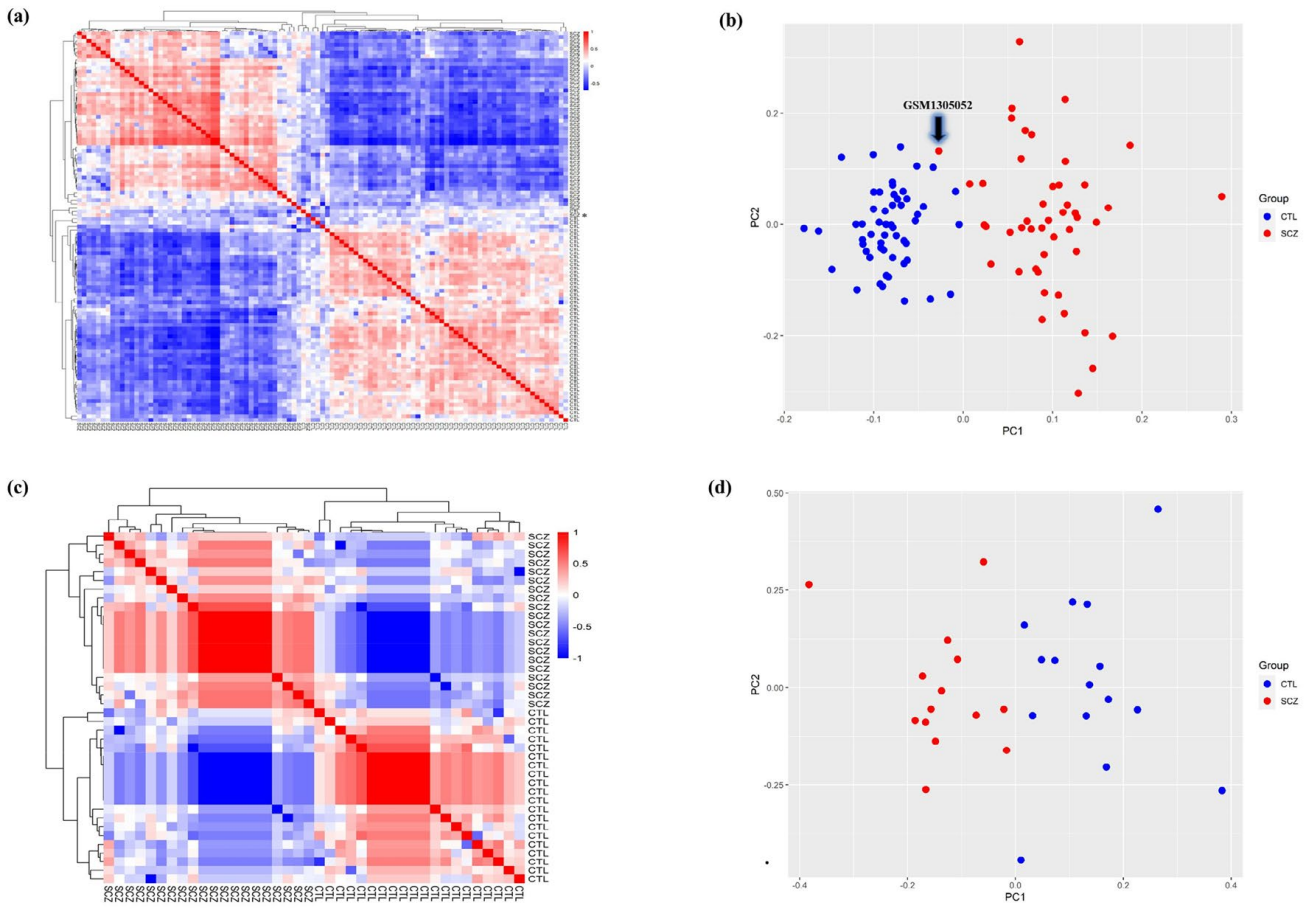
Hierarchically clustered correlation heatmap and principal component analysis (PCA) for the brain dataset (GSE53987) are shown in **(a)** and **(b)**, and for the lymphoblast dataset (GSE73129) are depicted in **(c)** and **(d)**, respectively. Each column in the heatmap represents one sample and shows the correlation to all samples (including itself), with red for correlation = 1 and blue for the lowest observed correlation. All SCZ and CTL samples correlated well with each other, except one SCZ sample from the GSE53987 study marked by an asterisk (*). In PCA, all samples are segregated by condition group (on PC1). GSM1305052 (a patient striatum sample) was removed from further analysis in order to its wrong spatial enrichment. This figure was made using R version 4.0.3 (https://www.r-project.org/). CTL, control; SCZ, schizophrenia.

**Figure 2:**
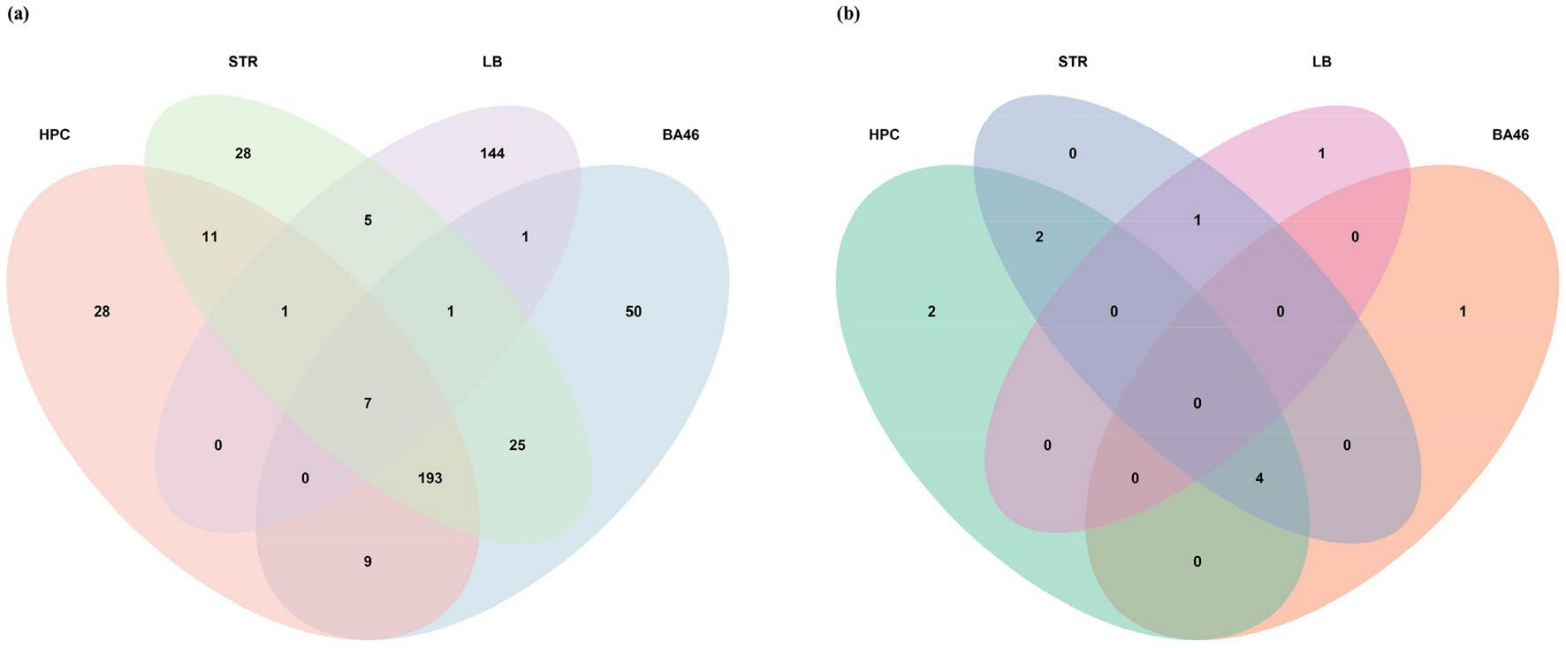
Analysis of differentially expressed genes (DEGs) in Schizophrenia (SCZ) brain and lymphoblast samples by Venn. Co-expression of **(a)** DEmRNAs and **(b)** DElncRNAs in different brain regions and lymphoblast of SCZ patients. This figure was made using R version 4.0.3 (https://www.r-project.org/). BA46, Brodmann area 46; HPC, hippocampus; LB, lymphoblast; STR, striatum.

Background correction, normalization, gene filtration, and batch correction were performed prior to DEGA. We applied AgiMicroRna Bioconductor package for quality control assessment. Box plots of gene expression data were depicted to evaluate data distribution following normalization (Supplementary Fig. S1). In the box plots, separate arrays had similar medians of expression level, confirming that correction was carried out properly. We checked sample correlation at the probe level. The samples were divided into two main clusters, one of which had SCZ, and the other consisted of the control samples. Moreover, a PCA plot was used to demonstrate the spatial distribution of samples (Fig. 1). PCA presents information about the structure of the analyzed data. It helps find similarities between samples. One of the STR samples in the SCZ group was removed due to being spatially far from other SCZ samples. The number of DEmRNAs and DElncRNAs are listed in Supplementary Table S1. Our analysis with the limma package in R identified 249 DEmRNAs (228 upregulated and 21 downregulated) (e.g., *BDNF*: brain-derived neurotrophic factor, *VEGFA*: vascular endothelial factor A, *FGF2*: fibroblast growth factor 2, *FOS*, and *CD44*), eight DElncRNAs (six upregulated and two downregulated) (e.g., *DLX6-AS1*: DLX6 antisense RNA 1, *NEAT1*: nuclear-enriched abundant transcript 1, *MINCR*: MYC-induced long non-coding RNA, *LINC01094*: long intergenic non-protein coding RNA 1094, *DLGAP1-AS1*: DLGAP1 antisense RNA 1, *BABAM2-AS1*: BABAM2 antisense RNA 1, and *PAX8-AS1*: PAX8 antisense RNA 1) in HPC region, 286 DEmRNAs (271 upregulated and 15 downregulated) (e.g., *VEGFA*, *FGF2*, *CD44*, *SOX2*: SRY-box transcription factor 2, and *NRAS*), five DElncRNAs (five upregulated) (including, *NEAT1*, *MINCR*, *LINC01094*, *PAX8-AS1*, and *ZFHX4-AS1*: ZFHX4 antisense RNA 1) in BA46 region, 271 DEmRNAs (250 upregulated and 21 downregulated) (e.g., *VEGFA, SPARC*: secreted protein acidic and rich in cysteine, *ZFP36*, *FGG*: fibrinogen gamma chain, and *ELAVL1*: ELAV-like protein 1), seven DElncRNAs (six upregulated and one downregulated) (e.g., *NEAT1*, *MINCR*, *LINC01094*, *DLGAP1-AS1*, *PAX8-AS1*, and *XIST*: X-inactive specific transcript) in STR region, 159 DEmRNAs (39 upregulated and 120 downregulated) (e.g., *STARD13*: StAR-related lipid transfer protein 13), and two lncRNAs (two upregulated) (e.g., *MALAT1*: metastasis associated lung adenocarcinoma transcript 1) in LB. Among the genes mentioned above, only *BDNF* and *DLX6-AS1* genes had decreased expression and others showed increased expression. The complete lists of DEGs are shown in Supplementary Tables S2–S5. Moreover, the Venn diagram indicated 15 DEmRNAs (*ADM*, *CLEC2B*, *F13A1*, *HLA-DQB1*, *HLA-DRB1*, *HLA-DRB3*, *HLA-DRB4*, *HLA-DRB5*, *CSF2RB*, *RHPN2*, *DDX3Y*, *EIF1AY*, *KDM5D*, *RPS4Y1*, *USP9Y*) and one DElncRNAs (*XIST*) were dysregulated in both brain and LB. The details of these overlapped genes are shown in Fig. 2 and Table 1.

**Table 1:** List of DEmRNAs and DElncRNAs that were dysregulated in both brain and blood.

Samples	DEmRNAs	DElncRNA
HPC and LB	*ADM*, *CLEC2B*, *F13A1*, *HLA-DQB1*, *HLA-DRB1*, *HLA-DRB3*, *HLA-DRB4*, *HLA-DRB5*	–
BA46 and LB	*ADM*, *CSF2RB*, *F13A1*, *HLA-DQB1*, *HLA-DRB1*, *HLA-DRB3*, *HLA-DRB4*, *HLA-DRB5*, *RHPN2*	–
STR and LB	*ADM*, *CLEC2B*, *CSF2RB*, *DDX3Y*, *EIF1AY*, *F13A1*, *HLA-DQB1*, *HLA-DRB1*, *HLA-DRB3*, *HLA-DRB4*, *HLA-DRB5*, *KDM5D*, *RPS4Y1*, *USP9Y*	*XIST*
HPC, BA46, and LB	*ADM*, *F13A1*, *HLA-DQB1*, *HLA-DRB1*, *HLA-DRB3*, *HLA-DRB4*, *HLA-DRB5*	–
HPC, STR, and LB	*ADM*, *CLEC2B*, *F13A1*, *HLA-DQB1*, *HLA-DRB1*, *HLA-DRB3*, *HLA-DRB4*, *HLA-DRB5*	–
BA46, STR, and LB	*ADM*, *CSF2RB*, *F13A1*, *HLA-DQB1*, *HLA-DRB1*, *HLA-DRB3*, *HLA-DRB4*, *HLA-DRB5*	–
HPC, BA46, STR, and LB	*ADM*, *F13A1*, *HLA-DQB1*, *HLA-DRB1*, *HLA-DRB3*, *HLA-DRB4*, *HLA-DRB5*	–

*BA46* Brodmann area 46, *DElncRNA* differentially expressed lncRNA, *DEmRNAs* differentially expressed mRNAs, *HPC* hippocampus, *LB* lymphoblast, *STR* striatum.

### lncRNA-associated ceRNA network construction

**Figure 3:**
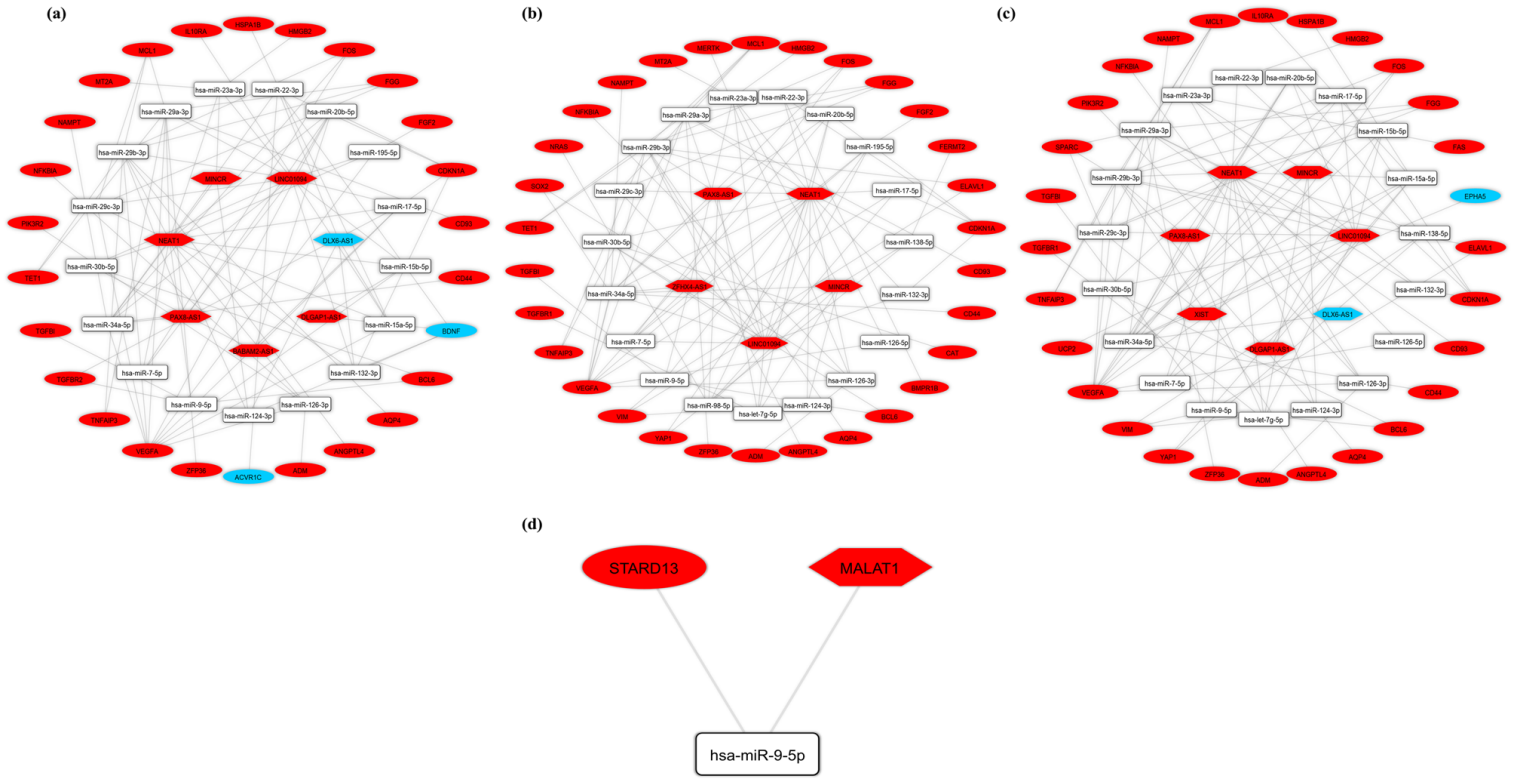
The long non-coding RNA-associated competing endogenous RNA (ceRNA) network. ceRNA axes in **(a)** hippocampus, **(b)** Brodmann area 46, **(c)** striatum regions and **(d)** lymphoblasts. The red and blue nodes represent the upregulation and downregulation, respectively. Gray edges represent interactions between RNAs. This figure was generated using Cytoscape version 3.8.0 (https://cytoscape.org/)^39^. LncRNAs, miRNAs, and mRNAs are represented by hexagon, round rectangle, and ellipse, respectively.

The DIANA-LncBase v3 online tool was used to anticipate DElncRNA-miRNA interaction pairings. DELncRNAs, targeted DEmRNAs, and also the interacted miRNAs were deleted from the ceRNA network in the opposite expression pattern present between DElncRNAs and the targeted DEmRNAs. Following that, miRTarBase was utilized to identify interactions between target mRNAs and SCZ-associated miRNAs (discovered by the HMDD). We developed a regulatory ceRNA network based on DElncRNA-miRNA-DEmRNA interactions to explore the potential mechanism behind SCZ pathophysiology (Fig. 3 and Table 2). CeRNA network contained 50 nodes (seven lncRNAs, 17 miRNAs, and 26 mRNAs) and 95 edges in HPC, 54 nodes (five lncRNAs, 19 miRNAs, and 30 mRNAs) and 101 edges in BA46, 54 nodes (seven lncRNAs, 19 miRNAs, and 28 mRNAs) and 97 edges in STR, and three nodes (one lncRNA, one miRNA, and one mRNA) and two edges in LB. There was no common ceRNA axis between LB and brain regions. We identified one ceRNA axis in LB (*MALAT1*/*hsa-miR-9-5p*/*STARD13*). Among the list, 90 different ceRNA axes were common between all three areas of the brain. These ceRNA axes are bolded in Table 1.

**Table 2:** Details of competing endogenous RNA (ceRNA) axes.

DElncRNA(s)	Shared miRNA(s)	DEmRNA(s)	Sample	Expression of DElncRNA(s) and DEmRNA(s)
***DLX6-AS1***	*hsa-miR-22-3p*	*ACVR1C, BDNF*	HPC	Down
***DLX6-AS1***	*hsa-miR-124-3p* *hsa-miR-132-3p* *hsa-miR-15a-5p*	*BDNF*	HPC	Down
***DLX6-AS1***	*hsa-miR-708-5p*	*NNAT*	HPC	Down
***BABAM2-AS1,**** NEAT1****,**** LINC01094*	***hsa-miR-30b-5p***	***BCL6***	HPC	Up
*NEAT1*	***hsa-miR-9-5p***	***BCL6****, ****TGFBI****, TGFBR2*	HPC	Up
*LINC01094****,**** NEAT1*	***hsa-miR-132-3p***	***CDKN1A***	HPC	Up
*NEAT1*	***hsa-miR-22-3p***	***CDKN1A***	HPC	Up
***BABAM2-AS1,**** LINC01094****,**** NEAT1*	***hsa-miR-17-5p***	***CDKN1A****, TGFBR2, ****VEGFA***	HPC	Up
*NEAT1****,**** LINC01094*	***hsa-miR-29a-3p***	***FGG****, TET1, ****VEGFA****, ****ZFP36****, ****TNFAIP3****, ****MCL1****, ****CD93***	HPC	Up
*LINC01094****,**** NEAT1*	***hsa-miR-7-5p***	***FOS***	HPC	Up
*NEAT1****,**** LINC01094*	***hsa-miR-29c-3p***	***MCL1****, ****FGG****, ****VEGFA***	HPC	Up
*LINC01094****,**** MINCR****,**** NEAT1*	***hsa-miR-23a-3p***	*MT2A, ****HMGB2***	HPC	Up
*NEAT1*	***hsa-miR-126-3p***	***NFKBIA****, PIK3R2, ****ADM****, ****VEGFA***	HPC	Up
*PAX8-AS1****,**** LINC01094****,**** NEAT1*	***hsa-miR-29b-3p***	*TET1, ****FOS****, ****ANGPTL4****, ****AQP4****, ****FGG****, ****VEGFA****, ****TNFAIP3****, ****MCL1***	HPC	Up
***BABAM2-AS1,**** PAX8-AS1****,**** LINC01094****,**** NEAT1*	***hsa-miR-20b-5p***	***VEGFA****, ****CDKN1A***	HPC	Up
*LINC01094****,**** NEAT1*	***hsa-miR-195-5p***	***VEGFA****, ****FGF2***	HPC	Up
*NEAT1****, DLGAP1-AS1***	***hsa-miR-15a-5p***	***VEGFA****, ****IL10RA****, ****HSPA1B***	HPC	Up
*PAX8-AS1****,**** LINC01094****,**** NEAT1****,**** MINCR*	***hsa-miR-34a-5p***	***VEGFA****, ****NAMPT****, ****FOS****, ****CD44***	HPC	Up
*NEAT1*	***hsa-miR-126-5p***	***VEGFA***	HPC	Up
*NEAT1****,**** LINC01094****, DLGAP1-AS1***	***hsa-miR-15b-5p***	***VEGFA***	HPC	Up
*NEAT1*	***hsa-miR-126-3p***	***ADM****, SOX2, ****VEGFA****, MERTK, ****NFKBIA***	BA46	Up
*NEAT1****,**** LINC01094*	***hsa-miR-30b-5p***	***BCL6****, CAT*	BA46	Up
*NEAT1*	***hsa-miR-22-3p***	***CDKN1A****, BMPR1B*	BA46	Up
*LINC01094****,**** NEAT1*	***hsa-miR-132-3p***	***CDKN1A***	BA46	Up
*NEAT1****,**** LINC01094*	***hsa-miR-17-5p***	***CDKN1A****, ****VEGFA***	BA46	Up
*PAX8-AS1****,**** LINC01094****,**** NEAT1*	***hsa-miR-20b-5p***	***CDKN1A****, ****VEGFA***	BA46	Up
***NEAT1***	*hsa-miR-138-5p*	*FERMT2, VIM, YAP1*	BA46	Up
***ZFHX4-AS1,**** LINC01094****,**** NEAT1*	***hsa-miR-7-5p***	***FOS***	BA46	Up
*NEAT1****,**** MINCR****,**** LINC01094****, ZFHX4-AS1***	***hsa-miR-23a-3p***	***HMGB2****, MT2A*	BA46	Up
*NEAT1****,**** LINC01094*	***hsa-miR-29a-3p***	***MCL1****, ****ZFP36****, TET1, ****FGG****, ****VEGFA****, ****TNFAIP3****, ****CD93***	BA46	Up
***NEAT1, PAX8-AS1, MINCR, LINC01094***	*hsa-miR-98-5p*	*NRAS*	BA46	Up
*MINCR****,**** PAX8-AS1****,**** LINC01094****, ZFHX4-AS1,**** NEAT1*	***hsa-miR-34a-5p***	*SOX2, ****VEGFA****, ****NAMPT****, ****CD44****, ****FOS***	BA46	Up
***NEAT1, PAX8-AS1, LINC01094, MINCR***	*hsa-let-7 g-5p*	*TGFBR1*	BA46	Up
*LINC01094****,**** NEAT1*	***hsa-miR-15b-5p***	***VEGFA***	BA46	Up
*LINC01094****,**** PAX8-AS1****,**** NEAT1*	***hsa-miR-29b-3p***	***VEGFA****, ****MCL1****, ELAVL1, ****TNFAIP3****, ****FOS****, ****FGG****, ****AQP4****, ****ANGPTL4****, TET1*	BA46	Up
*NEAT1****,**** LINC01094*	***hsa-miR-29c-3p***	***VEGFA****, ****MCL1****, ****FGG***	BA46	Up
*NEAT1*	***hsa-miR-126-5p***	***VEGFA***	BA46	Up
***ZFHX4-AS1,**** NEAT1*	***hsa-miR-9-5p***	*VIM, ****BCL6****, ELAVL1, ****TGFBI***	BA46	Up
***NEAT1, MINCR***	*hsa-miR-124-3p*	*VIM*	BA46	Up
*NEAT1****,**** LINC01094*	***hsa-miR-195-5p***	*YAP1, ****VEGFA****, ****FGF2***	BA46	Up
*NEAT1*	***hsa-miR-15a-5p***	*YAP1, ****VEGFA****, ****IL10RA****, UCP2, ****HSPA1B***	BA46	Up
***DLX6-AS1***	*hsa-miR-34a-5p*	*EPHA5*	STR	Down
*NEAT1*	***hsa-miR-126-3p***	***ADM****, ****VEGFA****, ****NFKBIA****, PIK3R2*	STR	Up
*LINC01094****,**** NEAT1*	***hsa-miR-30b-5p***	***BCL6***	STR	Up
*NEAT1*	***hsa-miR-22-3p***	***CDKN1A***	STR	Up
*NEAT1****,**** LINC01094*	***hsa-miR-132-3p***	***CDKN1A***	STR	Up
*MINCR****,**** LINC01094****,**** NEAT1*	***hsa-miR-23a-3p***	*FAS, ****HMGB2***	STR	Up
*NEAT1****,**** LINC01094*	***hsa-miR-7-5p***	***FOS***	STR	Up
*LINC01094****,**** MINCR, PAX8-AS1****,**** NEAT1*	***hsa-miR-34a-5p***	***FOS****, ****VEGFA****, ****NAMPT****, ****CD44***	STR	Up
*LINC01094****,**** NEAT1*	***hsa-miR-29a-3p***	***MCL1****, ****VEGFA****, ****TNFAIP3****, ****CD93****, SPARC, ****ZFP36****, ****FGG***	STR	Up
***MINCR, PAX8-AS1, LINC01094, NEAT1***	*hsa-let-7 g-5p*	*TGFBR1*	STR	Up
***DLGAP1-AS1,**** NEAT1*	***hsa-miR-15a-5p***	*UCP2, ****VEGFA****, ****IL10RA****, ****HSPA1B****, YAP1*	STR	Up
*NEAT1****,**** LINC01094*	***hsa-miR-17-5p***	***VEGFA****, ****CDKN1A***	STR	Up
*PAX8-AS1****,**** NEAT1****,**** LINC01094*	***hsa-miR-20b-5p***	***VEGFA****, ****CDKN1A***	STR	Up
***DLGAP1-AS1,**** LINC01094****,**** NEAT1*	***hsa-miR-15b-5p***	***VEGFA***	STR	Up
*LINC01094****,**** PAX8-AS1****,**** NEAT1*	***hsa-miR-29b-3p***	***VEGFA****, ****MCL1****, ELAVL1, SPARC, ****ANGPTL4****, ****AQP4****, ****FGG****, ****FOS****, ****TNFAIP3***	STR	Up
*LINC01094****,**** NEAT1****, XIST***	***hsa-miR-29c-3p***	***VEGFA****, ****MCL1****, ****FGG****, SPARC*	STR	Up
*NEAT1*	***hsa-miR-126-5p***	***VEGFA***	STR	Up
*LINC01094****,**** NEAT1*	***hsa-miR-195-5p***	***VEGFA****, YAP1, ****FGF2***	STR	Up
*NEAT1*	***hsa-miR-9-5p***	*VIM, ELAVL1, ****BCL6****, ****TGFBI***	STR	Up
***NEAT1, MINCR***	*hsa-miR-124-3p*	*VIM*	STR	Up
***NEAT1***	*hsa-miR-138-5p*	*VIM, YAP1*	STR	Up
***MALAT1***	*hsa-miR-9-5p*	*STARD13*	LB	Up

Bold values are common ceRNA axes between all three areas of the brain.

*BA46* Brodmann area 46, *DElncRNAs* differentially expressed lncRNAs, *DEmRNAs* differentially expressed mRNAs, *down* downregulation, *HPC* hippocampus, *LB* lymphoblast, *STR* striatum, *up* upregulation.

### Pathway enrichment analysis of DEmRNAs

**Figure 4:**
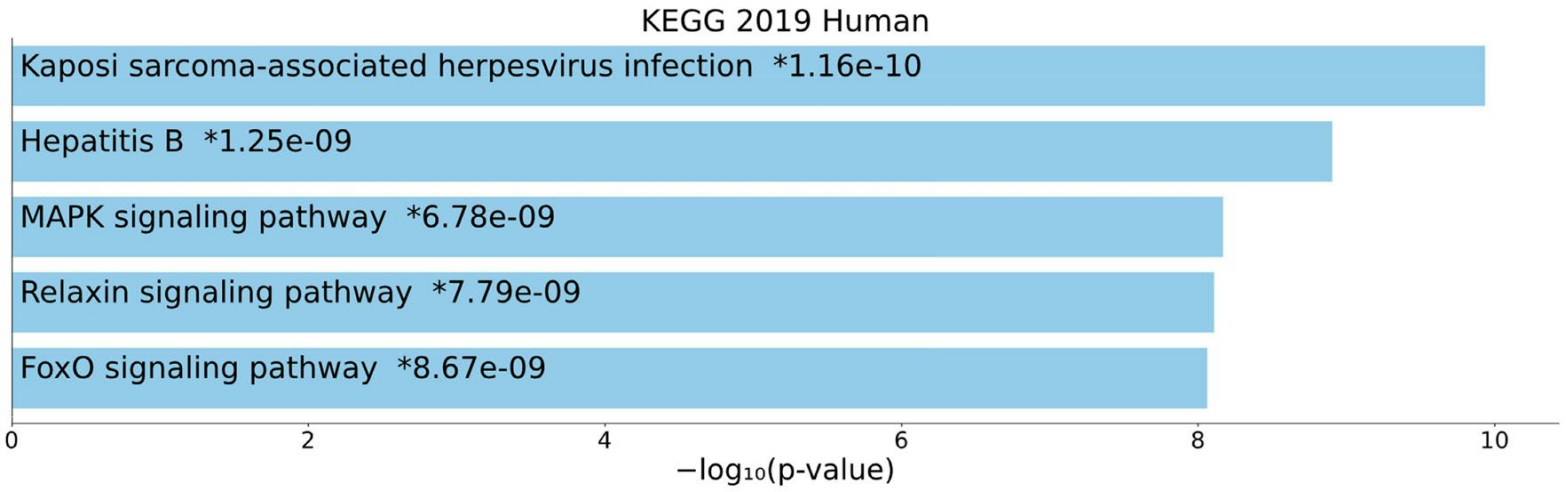
Overall results of pathway enrichment analysis. The bar chart shows the top five enriched pathways, along with their corresponding *P*-values. Colored bars correspond to terms with significant *P*-values (< 0.05). An asterisk (*) next to a *P*-value indicates the term also has a significant adjusted *P*-value (< 0.05). This chart was generated with Appyter (https://appyters.maayanlab.cloud/#/)^44^.

A KEGG enrichment analysis of all expressed DEmRNAs that were in the ceRNA network was conducted. The top five enriched KEGG pathways were “Kaposi sarcoma-associated herpesvirus infection”, “Hepatitis B”, “MAPK signaling pathway”, “Relaxin signaling pathway”, and “FoxO signaling pathway” (Fig. 4).

### PPI network construction and hub genes identification

**Figure 5:**
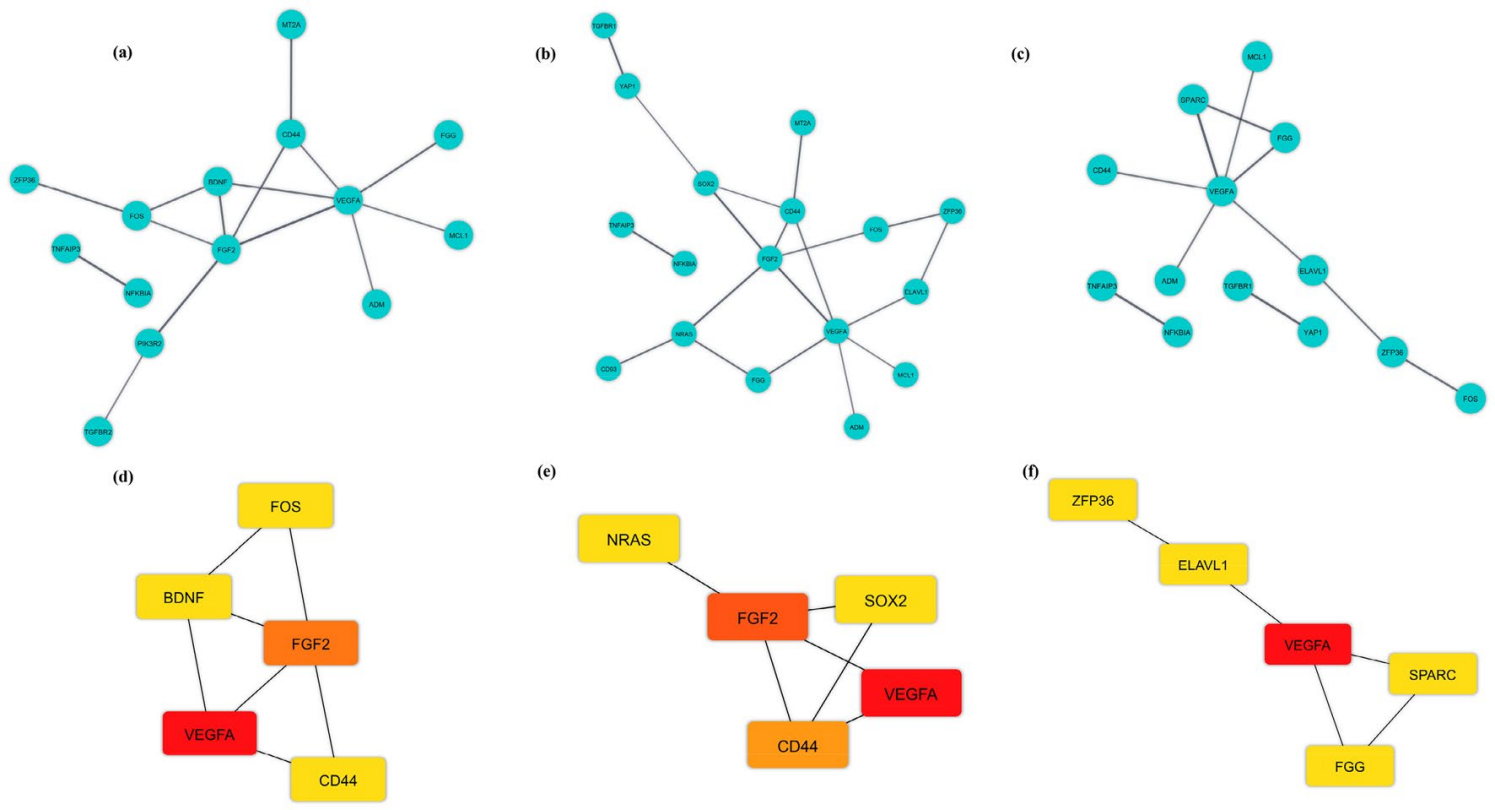
Protein–protein interaction (PPI) network of differentially expressed mRNAs (DEmRNAs) and hub genes in three brain regions. Blue nodes show the interaction among DEmRNAs in the PPI network in **(a)** hippocampus (HPC), **(b)** Brodmann area 46 (BA46), and **(c)** striatum (STR) regions. Hub genes in **(d)** HPC, **(e)** BA46, and **(f)** STR regions identified from the PPI network. Red, orange, and yellow nodes represent the hub genes with strong, moderate, and weak connections, respectively. This figure was generated using Cytoscape version 3.8.0 (https://cytoscape.org/)^39^.

The PPI network of three brain regions is shown in Fig. 5a–c. We assessed their degree of connection and recognized 11 hub genes in three brain regions (*BDNF*, *VEGFA*, *FGF2*, *FOS*, *CD44*, *SOX2*, *NRAS*, *SPARC*, *ZFP36*, *FGG*, and *ELAVL1*), which were all upregulated except *BDNF*. The strongest connections with others were observed for the hub genes marked with red nodes. In addition, orange and yellow nodes represent the hub genes with moderate and weak connections, respectively (Fig. 5d–f). *VEGFA* had the strongest connections with others in the HPC region, *FGF2* had moderate connections, while the hub genes *CD44*, *BDNF*, and *FOS* had weak connections. The *VEGFA* hub gene had the strongest connections with others in the BA46 region, whereas the hub genes *CD44* and *FGF2* had moderate connections, and the hub genes *NRAS* and *SOX2* had weak connections. The *VEGFA* gene exhibited the strongest connections with others in the STR region, whereas the hub genes *ELAVL1*, *SPARC*, *FGG*, and *ZFP36* had weak connections. Based on these findings, *VEGFA* had the strongest connections in all three regions of the brain. Two other genes (*FGF2* and *CD44*) were also identified as hub genes in both HPC and BA46 regions.

### Reconstruction of the subceRNA network

**Figure 6:**
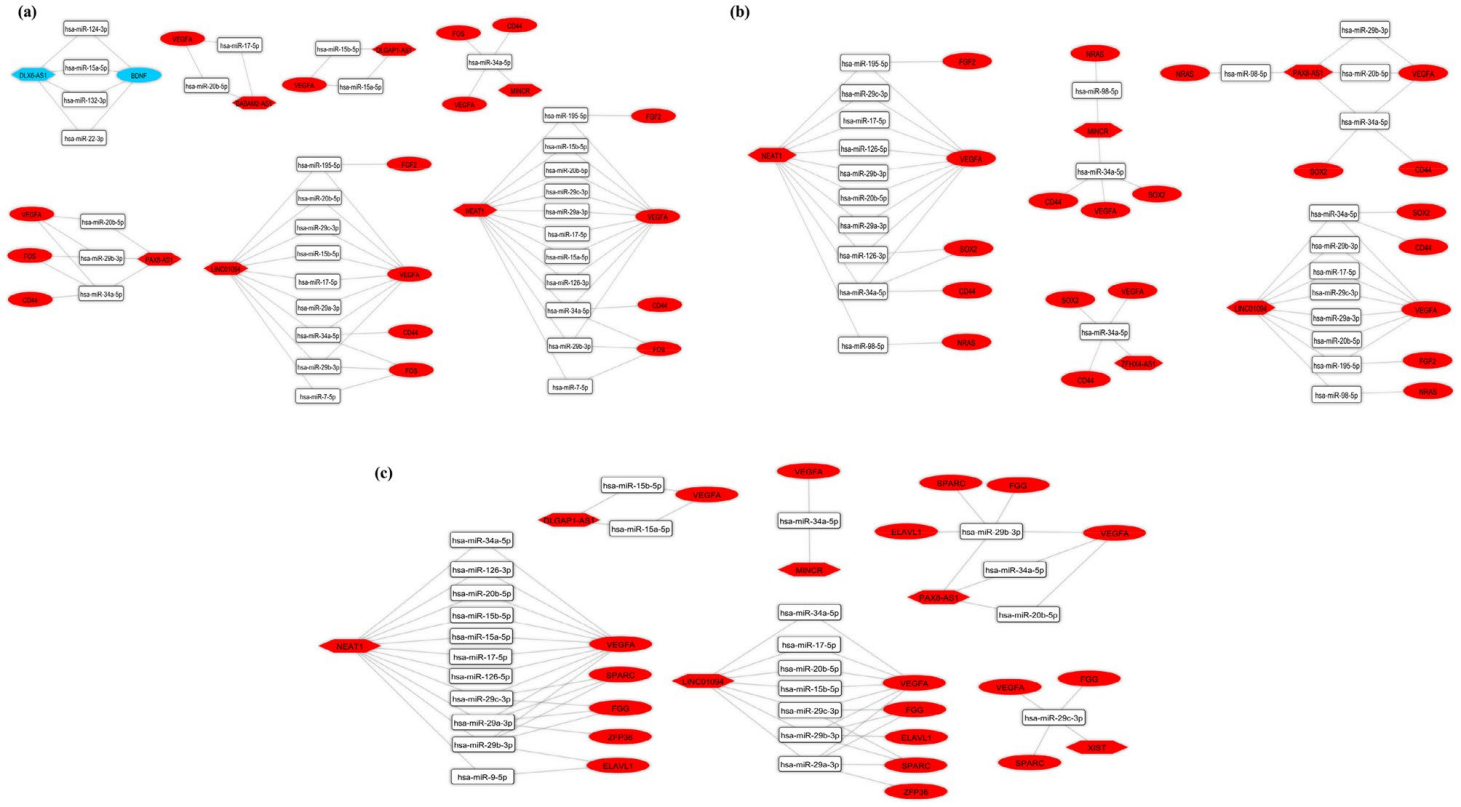
The lncRNA-miRNA-hub gene subceRNA network. ceRNA axes in **(a)** hippocampus, **(b)** Brodmann area 46, and **(c)** striatum regions. The red and blue nodes represent the upregulation and downregulation, respectively. Gray edges represent interactions between RNAs. LncRNAs, miRNAs, and mRNAs are represented by hexagon, round rectangle, and ellipse, respectively. This figure was generated using Cytoscape version 3.8.0 (https://cytoscape.org/)^39^. CeRNA, competing endogenous RNA.

Based on the hub genes, the lncRNA-miRNA-hub genes’ subnetwork of three brain regions was reconstructed. In total, nine lncRNAs (*DLX6-AS1*, *NEAT1*, *MINCR*, *LINC01094*, *DLGAP1-AS1*, *BABAM2-AS1*, *PAX8-AS1*, *ZFHX4-AS1*, and *XIST*), 17 miRNAs (*hsa-miR-124-3p*, *hsa-miR-132-3p*, *hsa-miR-15a-5p*, *hsa-miR-22-3p*, *hsa-miR-126-3p*, *hsa-miR-15b-5p*, *hsa-miR-17-5p*, *hsa-miR-195-5p*, *hsa-miR-20b-5p*, *hsa-miR-29a-3p*, *hsa-miR-29b-3p*, *hsa-miR-29c-3p*, *hsa-miR-34a-5p*, *hsa-miR-7-5p*, *hsa-miR-126-5p*, *hsa-miR-98-5p*, and *hsa-miR-9-5p*) and 11 hub genes (*BDNF*, *VEGFA*, *FGF2*, *FOS*, *CD44*, *SOX2*, *NRAS*, *SPARC*, *ZFP36*, *FGG*, and *ELAVL1*) were included (Fig. 6). We used “key lncRNAs”, “key miRNAs”, and “key mRNAs” keywords to refer to these transcripts, respectively. CeRNA axes in which the lncRNA *NEAT1* regulates the *VEGFA* via sponging different miRNAs (e.g., miR-29 family and miR-20b), as well as *MALAT1*/*hsa-miR-9-5p*/*STARD13* (the only axis identified in the LB), could be good candidates for future experimental studies in different brain regions and peripheral blood of SCZ patients, respectively.

## Discussion

The expression patterns of ceRNA differ depending on tissue-related, cellular, and subcellular circumstances. A network might consist of different types of ceRNAs, such as lncRNAs, circRNAs, pseudogenes, as well as mRNAs^45^. LncRNAs, one of the major forms of RNAs, are identified in the ceRNA machinery and perform a significant role in physiological and pathological cellular mechanisms. It has been revealed that they are implicated in a variety of mental disorders (e.g., AD, SCZ, and autism spectrum disorder (ASD))^46^. It is now well accepted that lncRNA expression varies by tissue, cellular types, and developmental levels. This tissue specificity, besides subcellular distributions, clearly indicates that lncRNA expression is tightly regulated^47^. According to the as-mentioned theoretical concepts, the ceRNA regulation network related to lncRNAs can play a crucial role in SCZ pathogenesis. In this study, we used a public database to download the expression profiles of four different types of SCZ tissues to evaluate the DElncRNAs and DEmRNAs in SCZ and normal tissues and then create a lncRNA-miRNA-mRNA regulatory network. We identified possible lncRNA-miRNA-mRNA axes in the pathogenesis of SCZ consisting of nine key lncRNAs, 17 key miRNAs, 11 key mRNAs in the brain, and one key lncRNA, one key miRNA, one key mRNA in LB.

LncRNA expression has been hypothesized to be dysregulated in SCZ and might be involved in the pathogenesis^48^. Among the key lncRNAs identified by our analysis, only the association of *NEAT1*, *MALAT1*, and *DLX6-AS1* with SCZ has been the specific focus of previous studies. *NEAT1* is a member of the lncRNA family highly enriched in the mammalian brain^49^. It is a crucial scaffolding unit as well as a structural determinant of paraspeckles^50^, which is a subnuclear structure implicated in several cellular processes such as splicing and transcriptional modulation through chromatin structure modification^49^. This type of lncRNA has already been found to be upregulated in other types of mental illnesses, namely Huntington’s disease^51^ and ASD^52^. Studies related to the comparison of *NEAT1* expression in the patients with SCZ against the control group have shown contradictory findings. Some studies identified downregulation of *NEAT1* in peripheral blood^53^ and multiple subareas of the cerebral cortex such as BA46 and HPC^54^ of SCZ patients compared with healthy controls. However, others revealed no significant difference in peripheral blood^18^, or upregulation in BA46, HPC, and STR regions^7^, which is similar to our results. Further studies are needed to explain these contradictory findings. *MALAT1* is predominantly expressed in nerve cells and is transcriptionally elevated in nuclear speckles. It has been reported that *MALAT1* employs splicing proteins of the Ser/Arg (SR) family in transcription sites in order to regulate synaptogenesis-related gene expression in hippocampal neurons cultivated. Furthermore, in this system, knocking down *MALAT1* results in a reduction in synaptic density, while overexpression promotes the density^55^. A study recently has shown no substantial variation in the expression level of *MALAT1* in SCZ patients’ peripheral blood compared with healthy control^56^. This finding is inconsistent with our results which might be attributed to the treatment of the patients with clozapine. The upregulation of *DLX6-AS1*, a regulator of GABAergic interneuron development^57^, in cerebral organoids derived from induced pluripotent stem cells showed that it might play a key role in ASD and SCZ pathogenesis^58^. In addition, we found *XIST* as another key lncRNA in the ceRNA network, which is associated with mental disorders. LncRNA *XIST* regulates the inactivation of the X chromosome, which is a critical developmental mechanism in the brain. *XIST* is highly expressed in lymphoblastoid cells from female patients diagnosed with bipolar disorder or recurrent severe depression as well as postmortem human brains of female psychiatric patients. Our results are in line with these findings. To our knowledge, the association of the other six key lncRNAs (*MINCR*, *LINC01094*, *DLGAP1-AS1*, *BABAM2-AS1*, *PAX8-AS1*, and *ZFHX4-AS1*) has not been the specific focus of investigations in the context of mental disorders. *MINCR* is a Myc-induced lncRNA related to some cancers via acting as a ceRNA^59^. Similarly, recent studies report that *LINC01094*^60^, *DLGAP1-AS1*^61^, and *PAX8-AS1*^62^ can act as a ceRNA in some cancers. Besides, it has been shown that *BABAM2-AS1* (also known as *BRE-AS1*)^63^ and *ZFHX4-AS1*^64^ regulate cancer cells through multiple mechanisms. The association between some of the lncRNAs with SCZ has been reported in this study for the first time; therefore, further studies are needed to validate our findings.

MiRNAs control target gene expression by attaching to the non-transcript site of the targeted gene, which then influences biological and signals transduction pathways in a cell, with potential effect on the onset and progression of SCZ^65^. In our work, we found that key lncRNAs may regulate hub genes via sponging 17 SCZ-associated key miRNAs (*hsa-miR-124-3p*, *hsa-miR-132-3p*, *hsa-miR-15a-5p*, *hsa-miR-22-3p*, *hsa-miR-126-3p*, *hsa-miR-15b-5p*, *hsa-miR-17-5p*, *hsa-miR-195-5p*, *hsa-miR-20b-5p*, *hsa-miR-29a-3p*, *hsa-miR-29b-3p*, *hsa-miR-29c-3p*, *hsa-miR-34a-5p*, *hsa-miR-7-5p*, *hsa-miR-126-5p*, *hsa-miR-98-5p*, and *hsa-miR-9-5p*). Our prediction is that most of these miRNAs show decreased expression, except for miRNAs that target hub genes that have reduced expression (e.g., *BDNF*). According to the HMDD database, some of these miRNAs, such as *miR-132*^66^, *miR-195*^67^, *miR-20b*, *miR-29* family^68^, are downregulated, while some others, such as *miR-15* family, *miR-126*^15^, *miR-17*^69^ are upregulated in some brain regions of SCZ patients. Although most of these findings can confirm our results, the predicted ceRNA axes must still be validated by molecular techniques such as luciferase reporter systems, co-immunoprecipitation assays, and PCR.

In the present study, KEGG enrichment analysis was performed as well. The results showed that most of the DEmRNAs involve in viral infection and three different signaling pathways. A previous study found a higher prevalence of human herpesvirus 8 (HHV8) infection in the patients with SCZ against the control group^70^. HHV8 has been associated with the development of Kaposi sarcoma^71^. Previously, a meta-analysis demonstrated that people with SCZ are more likely to have infectious hepatitis, which could be attributed to behavioral, immunological parameters, or both^72^. It is also shown that expression levels of mitogen-activated protein kinase (MAPK)^73^ and forkhead box O (FOXO) pathway-related genes^74^ are altered in SCZ. Association between relaxin polymorphisms and SCZ also suggests that the relaxin pathway plays a role in the development of SCZ^75^. Dysfunction of these signaling pathways might lead to functional pathology in SCZ.

We found that 15 mRNAs (*ADM*, *CLEC2B*, *F13A1*, *HLA-DQB1*, *HLA-DRB1*, *HLA-DRB3*, *HLA-DRB4*, *HLA-DRB5*, *CSF2RB*, *RHPN2*, *DDX3Y*, *EIF1AY*, *KDM5D*, *RPS4Y1*, *USP9Y*) and one lncRNA (*XIST*) were differentially expressed in the LB and brain of patients with SCZ using a Venn diagram, suggesting that these RNAs could be regarded as a biomarker for SCZ. Among them, *ADM*^76^ and *HLA-DRB1*^77^ have been suggested as biomarkers in previous studies.

Twelve key mRNAs (*BDNF*, *VEGFA*, *FGF2*, *FOS*, *CD44*, *SOX2*, *NRAS*, *SPARC*, *ZFP36*, *FGG*, *ELAVL1*, and *STARD13*) were reported in this study. BDNF is capable of regulating neuroplasticity, inhibiting the cascade of apoptosis, and raising the expression of many cellular proteins essential for neurogenesis, neuronal proliferation, as well as survival^78^. A myriad of studies have shown that low *BDNF* levels are associated with poor cognitive skills in SCZ patients^79^, which is consistent with our result. The VEGFA molecule is necessary for angiogenesis, brain plasticity, and neurogenesis, and it differs from other neurotrophic factors due to its significant angiogenic role. VEGFA can enhance the proliferation of neural stem cells, endothelial cells, mature astrocytes, and neurons. VEGF signaling is changed in SCZ, according to the results from transcriptomic, epigenomic, and genetic variation studies in hippocampus and prefrontal cortex samples^80^. In addition, FGF2 is involved in angiogenesis. FGF2 was later confirmed to be a neurotrophic factor, significant in neural development and central nervous system maintenance. Serum levels of FGF2 protein were found to be substantially higher in patients with first-episode, drug-free SCZ^81^. As one of the immediate early genes most studied in the brain, *FOS* is believed to play a significant role in SCZ pathophysiology. In line with our findings, *FOS* is upregulated in some areas of an SCZ rat model brain^82^. Contrarily, some research found that *FOS* is highly expressed in non-neural peripheral specimens in addition to the reduction of the *FOS* in the brain of SCZ patients^83^. More research is required to understand these conflicting findings. Also, contradictory results have been obtained from the study of *CD44*^84–86^ and *SPARC*^87^ dysregulation components of the brain extracellular matrix, in some brain regions. Another key gene in our study was *SOX2*. The association of this gene, which is a promoter of neurogenesis, with SCZ has been investigated in some animal studies^88,89^. The *NRAS*-encoded protein, a Ras superfamily intrinsic GTPase, is commonly associated with cancer progression and advancement. It is also essential in neurodevelopmental disorders due to its involvement in signal transduction from activated receptors toward the MAPK cascade. A previous study reported higher expression of this gene in the frontal cortex of SCZ patients^90^. This data is in agreement with our results. The genetic network of hemostatic procedure demonstrated that *FGG*, as a coagulation-related mediator gene, may have a role in SCZ^91^. *ELAVL1* plays a crucial role in mRNA stabilization (mediated by AU-rich elements) and translation as well as neuronal mRNA post-transcriptional regulation. The capability of ELAVL1 in stabilizing mRNA targets may be influenced by posttranslational modifications. It is postulated that an inefficient regulation of MeCP2 transcription in the SZ cohort is caused by alterations in one of its interactant targets, ELAVL1^92^. To the best of our knowledge, the role of two other key genes (i.e., *ZFP36* and *STARD13*) has not been investigated in SCZ thus far. Similar to *ELAVL1*, *ZFP36* is disease-relevant RNA-binding proteins (RBPs) that interact with AU-rich sequences, but unlike *ELAVL1*, *ZFP36* mediates the decay of target mRNAs^93^. In addition, its association with mental conditions (e.g., AD^94^ and suicidal behavior^95^) has been reported previously. Moreover, *STARD13*, a GTPase-activating protein (GAP), inhibits RhoA and Cdc42 specifically in glioblastoma cells and other types of tumor models^96^.

There are some limitations in the present study. First, variations in methodology and the preparation of samples, the platform, patient characteristics, and data processing may all have an effect on gene expression profiles^97–99^. Secondly, it is challenging to explain dynamic variations in disease progression and the emergence of complications using postmortem brains^13^. Finally, a validation analysis is needed to substantiate the reliability of our findings.

## Conclusion

In conclusion, this study identified a lncRNA-associated ceRNA network potentially relevant for SCZ. This network includes key mRNAs (including *BDNF*, *VEGFA*, *FGF2*, *FOS*, *CD44*, *SOX2*, *NRAS*, *SPARC*, *ZFP36*, *FGG*, *ELAVL1*, and *STARD13*), and lncRNAs (*DLX6-AS1*, *NEAT1*, *MINCR*, *LINC01094*, *DLGAP1-AS1*, *BABAM2-AS1*, *PAX8-AS1*, *ZFHX4-AS1*, and *XIST*). While the functions of this network need to be investigated, the present study provides potential research targets to study molecular mechanisms which could be relevant for the pathogenesis of SCZ.

## Supplementary Information

Supplementary Figure 1.

Supplementary Table 1.

Supplementary Table 2.

Supplementary Table 3.

Supplementary Table 4.

Supplementary Table 5.

## References

[1] Marder SR, Cannon TD (2019). Schizophrenia. N. Engl. J. Med..

[2] Keshavan MS (2020). Neuroimaging in Schizophrenia. Neuroimaging Clin. N. Am..

[3] McCutcheon RA, Reis Marques T, Howes OD (2020). Schizophrenia: An overview. PJAMA Psychiatry.

[4] Narayan S (2008). Molecular profiles of schizophrenia in the CNS at different stages of illness. Brain Res..

[5] Roy M (2018). Proteomic analysis of postsynaptic proteins in regions of the human neocortex. Nat. Neurosci..

[6] Ramaker RC (2017). Post-mortem molecular profiling of three psychiatric disorders. Genome Med..

[7] Lanz TA (2019). Postmortem transcriptional profiling reveals widespread increase in inflammation in schizophrenia: A comparison of prefrontal cortex, striatum, and hippocampus among matched tetrads of controls with subjects diagnosed with schizophrenia, bipolar or major depressive disorder. Transl. Psychiatry.

[8] Lai CY (2016). Biomarkers in schizophrenia: A focus on blood based diagnostics and theranostics. World J. Psychiatry.

[9] Perkovic MN (2017). Theranostic biomarkers for schizophrenia. Int. J. Mol. Sci..

[10] Harris LW (2012). Comparison of peripheral and central schizophrenia biomarker profiles. PLoS ONE.

[11] Sullivan PF, Fan C, Perou CM (2006). Evaluating the comparability of gene expression in blood and brain. Am. J. Med. Genet. B.

[12] Vawter MP, Philibert R, Rollins B, Ruppel PL, Osborn TW (2017). Exon array biomarkers for the differential diagnosis of schizophrenia and bipolar disorder. Complex Psychiatry.

[13] Horiuchi Y (2016). Molecular signatures associated with cognitive deficits in schizophrenia: A study of biopsied olfactory neural epithelium. Transl. Psychiatry.

[14] Gibbons A, Udawela M, Dean B (2018). Non-coding RNA as novel players in the pathophysiology of schizophrenia. Noncoding RNA.

[15] Beveridge NJ, Gardiner E, Carroll AP, Tooney PA, Cairns MJ (2010). Schizophrenia is associated with an increase in cortical microRNA biogenesis. Mol. Psychiatry.

[16] Santarelli DM, Beveridge NJ, Tooney PA, Cairns MJ (2011). Upregulation of dicer and microRNA expression in the dorsolateral prefrontal cortex Brodmann area 46 in schizophrenia. Biol. Psychiatry.

[17] Meng Q (2018). The DGCR5 long noncoding RNA may regulate expression of several schizophrenia-related genes. Sci. Transl. Med..

[18] Safari MR, Komaki A, Arsang-Jang S, Taheri M, Ghafouri-Fard S (2019). Expression pattern of long non-coding RNAs in schizophrenic patients. Cell Mol. Neurobiol..

[19] Horváth S, Janka Z, Mirnics K (2011). Analyzing schizophrenia by DNA microarrays. Biol. Psychiat..

[20] Gejman PV, Sanders AR, Duan J (2010). The role of genetics in the etiology of schizophrenia. Psychiatr. Clin. North Am..

[21] Salmena L, Poliseno L, Tay Y, Kats L, Pandolfi PP (2011). A ceRNA hypothesis: The Rosetta Stone of a hidden RNA language?. Cell.

[22] Ghafouri-Fard S (2021). A review on the expression pattern of non-coding RNAs in patients with schizophrenia: With a special focus on peripheral blood as a source of expression analysis. Front. Psych..

[23] Ala U (2020). Competing endogenous RNAs, non-coding RNAs and diseases: An intertwined story. Cells.

[24] Lang Y, Zhang J, Yuan Z (2019). Construction and dissection of the ceRNA-ceRNA network reveals critical modules in depression. Mol. Med. Rep..

[25] Li Z (2020). Circular RNA in schizophrenia and depression. Front. Psychiatry.

[26] Irizarry RA (2003). Exploration, normalization, and summaries of high density oligonucleotide array probe level data. Biostatistics (Oxford, England).

[27] von Heydebreck A, Huber W, Gentleman R (2005). Encyclopedia of Genetics, Genomics, Proteomics and Bioinformatics.

[28] Lopez-Romero P (2020). AgiMicroRna: Processing and differential expression analysis of agilent microRNA chips. BMC Genom..

[29] Yeung KY, Ruzzo WL (2001). Principal component analysis for clustering gene expression data. Bioinformatics (Oxford, England).

[30] Wickham H (2016). ggplot2: Elegant Graphics for Data Analysis.

[31] Kolde, R. *pheatmap: Pretty Heatmaps*. (2019).

[32] Ritchie ME (2015). limma powers differential expression analyses for RNA-sequencing and microarray studies. Nucleic Acids Res..

[33] Huber W (2015). Orchestrating high-throughput genomic analysis with Bioconductor. Nat. Methods.

[34] Dashti S, Taheri M, Ghafouri-Fard S (2020). An in-silico method leads to recognition of hub genes and crucial pathways in survival of patients with breast cancer. Sci. Rep..

[35] Braschi B (2019). Genenames.org: The HGNC and VGNC resources in 2019. Nucleic Acids Res..

[36] Karagkouni D (2020). DIANA-LncBase v3: Indexing experimentally supported miRNA targets on non-coding transcripts. Nucleic Acids Res..

[37] Huang Z (2019). HMDD v3.0: A database for experimentally supported human microRNA-disease associations. Nucleic Acids Res..

[38] Huang HY (2020). miRTarBase 2020: Updates to the experimentally validated microRNA-target interaction database. Nucleic Acids Res..

[39] Shannon P (2003). Cytoscape: A software environment for integrated models of biomolecular interaction networks. Genome Res..

[40] Chen EY (2013). Enrichr: Interactive and collaborative HTML5 gene list enrichment analysis tool. BMC Bioinform..

[41] Kuleshov MV (2016). Enrichr: A comprehensive gene set enrichment analysis web server 2016 update. Nucleic Acids Res..

[42] Szklarczyk D (2019). STRING v11: Protein-protein association networks with increased coverage, supporting functional discovery in genome-wide experimental datasets. Nucleic Acids Res..

[43] Chin C-H (2014). cytoHubba: Identifying hub objects and sub-networks from complex interactome. BMC Syst. Biol..

[44] Clarke DJB (2021). Appyters: Turning Jupyter Notebooks into data-driven web apps. Patterns.

[45] Cai Y, Wan J (2018). Competing endogenous RNA regulations in neurodegenerative disorders: Current challenges and emerging insights. Front. Mol. Neurosci..

[46] Zuo L (2016). Long noncoding RNAs in psychiatric disorders. Psychiatr. Genet..

[47] Gloss BS, Dinger ME (2016). The specificity of long noncoding RNA expression. Biochim. Biophys. Acta.

[48] Wang Z, Tong Q, Liao H, Rao S, Huang X (2018). Long non-coding RNAs in schizophrenia. Neurol. Psychiatry Brain Res..

[49] West JA (2014). The long noncoding RNAs NEAT1 and MALAT1 bind active chromatin sites. Mol. Cell.

[50] Hirose T (2014). NEAT1 long noncoding RNA regulates transcription via protein sequestration within subnuclear bodies. Mol. Biol. Cell.

[51] Sunwoo JS (2017). Altered expression of the long noncoding RNA NEAT1 in Huntington's disease. Mol. Neurobiol..

[52] Sayad A, Omrani MD, Fallah H, Taheri M, Ghafouri-Fard S (2019). Aberrant expression of long non-coding RNAs in peripheral blood of autistic patients. J. Mol. Neurosci..

[53] Li J (2018). Relationship between schizophrenia and changes in the expression of the long non-coding RNAs Meg3, Miat, Neat1 and Neat2. J. Psychiatr. Res..

[54] Katsel P (2019). The expression of long noncoding RNA NEAT1 is reduced in schizophrenia and modulates oligodendrocytes transcription. NPJ Schizophr..

[55] Bernard D (2010). A long nuclear-retained non-coding RNA regulates synaptogenesis by modulating gene expression. Embo. J..

[56] Fallah H (2019). Sex-specific up-regulation of lncRNAs in peripheral blood of patients with schizophrenia. Sci. Rep..

[57] Berghoff EG (2013). Evf2 (Dlx6as) lncRNA regulates ultraconserved enhancer methylation and the differential transcriptional control of adjacent genes. Development.

[58] Wang P (2017). CRISPR/Cas9-mediated heterozygous knockout of the autism gene CHD8 and characterization of its transcriptional networks in cerebral organoids derived from iPS cells. Mol. Autism.

[59] Li Z, Xie X, Fan X, Li X (2020). Long non-coding RNA MINCR regulates miR-876-5p/GSPT1 axis to aggravate glioma progression. Neurochem. Res..

[60] Li XX, Yu Q (2020). Linc01094 accelerates the growth and metastatic-related traits of glioblastoma by sponging miR-126-5p. Oncol. Targets Ther..

[61] Liu L, Li X, Shi Y, Chen H (2021). Long noncoding RNA DLGAP1-AS1 promotes the progression of glioma by regulating the miR-1297/EZH2 axis. Aging.

[62] Zhang D, An X, Yu H, Li Z (2021). The regulatory effect of 6-TG on lncRNA-miRNA-mRNA ceRNA network in triple-negative breast cancer cell line. Biosci. Rep..

[63] Chen Z, Zhen M, Zhou J (2019). LncRNA BRE-AS1 interacts with miR-145-5p to regulate cancer cell proliferation and apoptosis in prostate carcinoma and has early diagnostic values. Biosci. Rep..

[64] Li SY (2019). Down-regulated long non-coding RNA RNAZFHX4-AS1 suppresses invasion and migration of breast cancer cells via FAT4-dependent Hippo signaling pathway. Cancer Gene Ther..

[65] Caputo V, Ciolfi A, Macri S, Pizzuti A (2015). The emerging role of MicroRNA in schizophrenia. CNS Neurol. Disord. Drug Targets.

[66] Miller BH (2012). MicroRNA-132 dysregulation in schizophrenia has implications for both neurodevelopment and adult brain function. Proc. Natl. Acad. Sci. U S A.

[67] Mellios N (2009). Molecular determinants of dysregulated GABAergic gene expression in the prefrontal cortex of subjects with schizophrenia. Biol. Psychiatry.

[68] Perkins DO (2007). microRNA expression in the prefrontal cortex of individuals with schizophrenia and schizoaffective disorder. Genome Biol..

[69] Wong J (2013). Expression of NPAS3 in the human cortex and evidence of its posttranscriptional regulation by miR-17 during development, with implications for schizophrenia. Schizophr. Bull..

[70] Hannachi N (2014). High prevalence of Human Herpesvirus 8 in schizophrenic patients. Psychiatry Res..

[71] Cannon MJ, Laney AS, Pellett PE (2003). Human herpesvirus 8: Current issues. Clin. Infect. Dis..

[72] Lluch E, Miller BJ (2019). Rates of hepatitis B and C in patients with schizophrenia: A meta-analysis. Gen. Hosp. Psychiatry.

[73] Deane AR, Potemkin N, Ward RD (2021). Mitogen-activated protein kinase (MAPK) signalling corresponds with distinct behavioural profiles in a rat model of maternal immune activation. Behav. Brain Res..

[74] Gu S, Cui F, Yin J, Fang C, Liu L (2021). Altered mRNA expression levels of autophagy- and apoptosis-related genes in the FOXO pathway in schizophrenia patients treated with olanzapine. Neurosci. Lett..

[75] Munro J (2012). Relaxin polymorphisms associated with metabolic disturbance in patients treated with antipsychotics. J. Psychopharmacol..

[76] Kakiuchi C (2008). Up-regulation of ADM and SEPX1 in the lymphoblastoid cells of patients in monozygotic twins discordant for schizophrenia. Am. J. Med. Genet..

[77] Mohammadi A, Rashidi E, Amooeian VG (2018). Brain, blood, cerebrospinal fluid, and serum biomarkers in schizophrenia. Psychiatry Res..

[78] Lu B, Nagappan G, Lu Y (2014). BDNF and synaptic plasticity, cognitive function, and dysfunction. Handb. Exp. Pharmacol..

[79] Pan S (2021). The microRNA-195 - BDNF pathway and cognitive deficits in schizophrenia patients with minimal antipsychotic medication exposure. Transl. Psychiatry.

[80] Lizano P (2018). VEGFA GENE variation influences hallucinations and frontotemporal morphology in psychotic disorders: A B-SNIP study. Transl. Psychiatry.

[81] Li XS (2018). Increased serum FGF2 levels in first-episode, drug-free patients with schizophrenia. Neurosci. Lett..

[82] Monfil T (2018). Hyper-response to novelty increases c-Fos expression in the hippocampus and prefrontal cortex in a rat model of schizophrenia. Neurochem. Res..

[83] Huang J (2019). Central and peripheral changes in FOS expression in schizophrenia based on genome-wide gene expression. Front. Genet..

[84] Pantazopoulos H (2020). Molecular signature of extracellular matrix pathology in schizophrenia. Eur. J. Neurosci..

[85] Fillman SG (2013). Increased inflammatory markers identified in the dorsolateral prefrontal cortex of individuals with schizophrenia. Mol. Psychiatry.

[86] Fillman SG, Cloonan N, Miller LC, Weickert CS (2013). Markers of inflammation in the prefrontal cortex of individuals with schizophrenia. Mol. Psychiatry.

[87] Rodrigues-Amorim D (2021). Changes in the brain extracellular matrix composition in schizophrenia: A pathophysiological dysregulation and a potential therapeutic target. Cell Mol. Neurobiol..

[88] Ferreira FR, de Moura NSB, Hassib L, Pombo TR (2020). Resveratrol ameliorates the effect of maternal immune activation associated with schizophrenia in adulthood offspring. Neurosci. Lett..

[89] Večeřa J (2018). HDAC1 and HDAC3 underlie dynamic H3K9 acetylation during embryonic neurogenesis and in schizophrenia-like animals. J. Cell Physiol..

[90] Bryzgalov LO (2018). Novel functional variants at the GWAS-implicated loci might confer risk to major depressive disorder, bipolar affective disorder and schizophrenia. BMC Neurosci..

[91] Huang K-C, Yang K-C, Lin H, Tsao TT-H, Lee S-A (2014). Transcriptome alterations of mitochondrial and coagulation function in schizophrenia by cortical sequencing analysis. BMC Genom..

[92] Bakshi K, Kemether EM (2020). Two thalamic regions screened using laser capture microdissection with whole human genome microarray in schizophrenia postmortem samples. Schizophr. Res. Treat..

[93] Mukherjee N (2014). Global target mRNA specification and regulation by the RNA-binding protein ZFP36. Genome Biol..

[94] Alkallas R, Fish L, Goodarzi H, Najafabadi HS (2017). Inference of RNA decay rate from transcriptional profiling highlights the regulatory programs of Alzheimer's disease. Nat. Commun..

[95] Calati R (2014). Influence of differentially expressed genes from suicide post-mortem study on personality traits as endophenotypes on healthy subjects and suicide attempters. Eur. Arch. Psychiatry Clin. Neurosci..

[96] Nicolas S, Abdellatef S, Haddad MA, Fakhoury I, El-Sibai M (2019). Hypoxia and EGF stimulation regulate VEGF expression in human glioblastoma multiforme (GBM) cells by differential regulation of the PI3K/Rho-GTPase and MAPK pathways. Cells.

[97] Mistry M, Pavlidis P (2010). A cross-laboratory comparison of expression profiling data from normal human postmortem brain. Neuroscience.

[98] Kumarasinghe N, Tooney PA, Schall U (2012). Finding the needle in the haystack: A review of microarray gene expression research into schizophrenia. Aust. N. Z. J. Psychiatry.

[99] Mistry M, Gillis J, Pavlidis P (2013). Genome-wide expression profiling of schizophrenia using a large combined cohort. Mol Psychiatry.

