# Supplementary material for: Bioinformatics analysis of long non-coding RNA-associated competing endogenous RNA network in schizophrenia
Source: Sci Rep. 2021 Dec 24;11:24413. doi: 10.1038/s41598-021-03993-3 (PMC8709859; doi:10.1038/s41598-021-03993-3)
Supplement: Supplementary file 1 — Supplementary Figure 1. [file 41598_2021_3993_MOESM1_ESM.docx]

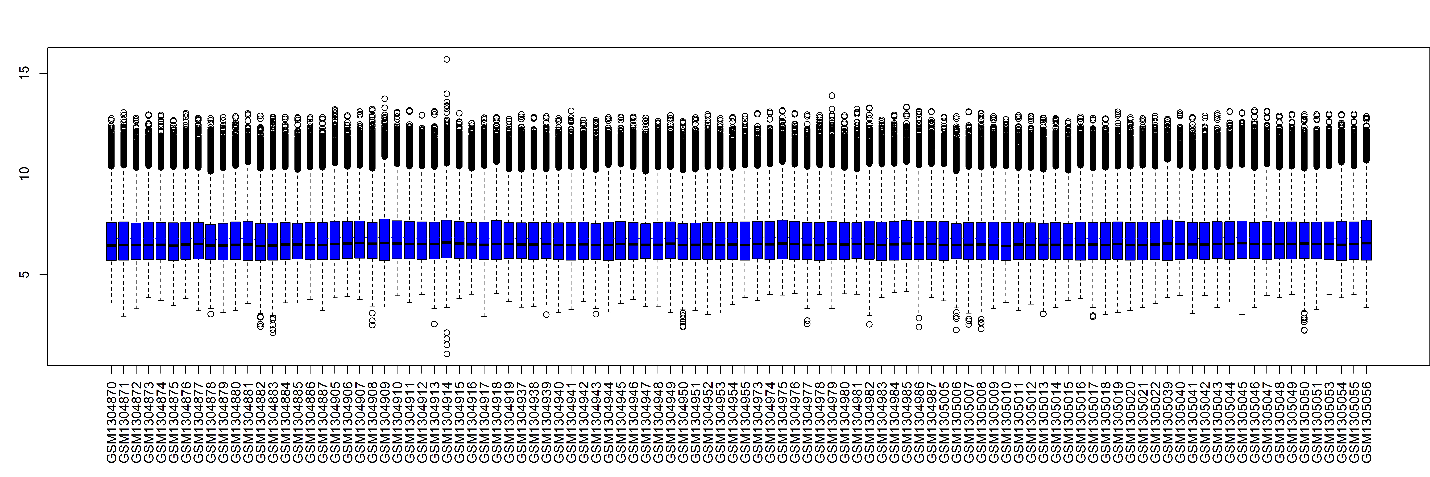


**(a)**


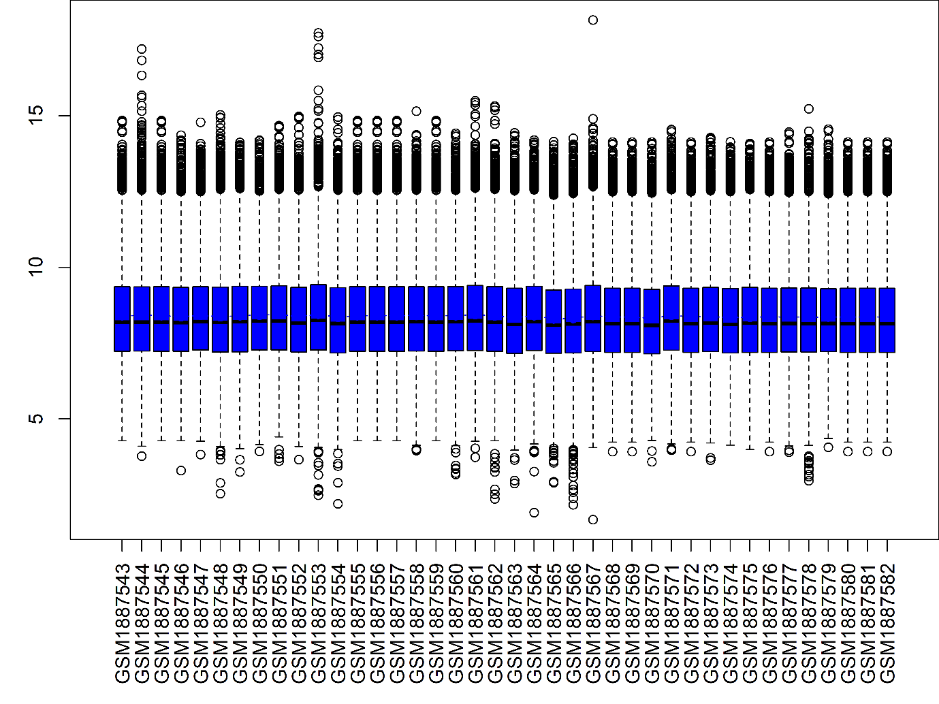


**(b)**

**Figure S1**. Boxplot for **(a)** GSE53987 study and **(b)** GSE73129 study. The horizontal axis represents the names of samples, and the vertical axis represents the lncRNA/mRNA expression.
