# Supplementary material for: Bioinformatics analysis of long non-coding RNA-associated competing endogenous RNA network in schizophrenia
Source: Sci Rep. 2021 Dec 24;11:24413. doi: 10.1038/s41598-021-03993-3 (PMC8709859; doi:10.1038/s41598-021-03993-3)
Supplement: Supplementary file 2 — Supplementary Table 1. [file 41598_2021_3993_MOESM2_ESM.docx]

| Table S1 Number of DEmRNAs and DElncRNAs in two studies | | | | | | | |
| --- | --- | --- | --- | --- | --- | --- | --- |
|  | DEmRNAs | | |  | DElncRNAs | | |
| Study | Total number of genes | Up* | Down** |  | Total number of genes | Up | Down |
| GSE53987  HPC region  BA46 region  STR region | 249  286  271 | 228  271  250 | 21  15  21 |  | 8  5  7 | 6  5  6 | 2  0  1 |
| GSE73129 | 159 | 39 | 120 |  | 2 | 2 | 0 |
| *, number of upregulated genes; **, number of downregulated genes; BA46, Brodmann area 46; DElncRNAs, differentially expressed lncRNAs; DEmRNAs, differentially expressed mRNAs; HPC, hippocampus; STR, striatum | | | | | | | |
